# Diabetic Foot Ulcers Combination with Lower Limb Lymphedema Treated by Staged Charles Procedure: Case Report and Literature Review

**DOI:** 10.12669/pjms.294.3479

**Published:** 2013

**Authors:** Chin-Ta Lin, Kuang-Wen Ou, Shun-Cheng Chang

**Affiliations:** 1 **Chin-Ta Lin, Division of Plastic and Reconstructive Surgery, Department of Surgery, Tri-Service General Hospital, National Defense Medical Center, Taipei, Taiwan.**; 2 **Kuang-Wen Ou, Division of Plastic and Reconstructive Surgery, Department of Surgery, Tri-Service General Hospital, National Defense Medical Center, Taipei, Taiwan.**; 3 **Shun-Cheng Chang, Division of Plastic and Reconstructive Surgery, Department of Surgery, Tri-Service General Hospital, National Defense Medical Center, Taipei, Taiwan.**

**Keywords:** Diabetic Foot, Ulcer, Charles procedure, Lymphedema

## Abstract

Primary or secondary, lymphedema is lymphatic dysfunction which results in protein-rich interstitial fluid accumulated in the skin and subcutaneous tissue. In developed countries, surgical resection of regional lymph nodes or chronic inflammation process is the most common etiology of lymphedema instead of parasite infection seen in developing countries. Patients with lymphedema sustain either cosmetic or functional problems, and several studies have indicated the potential risk, though not high, transforming lymphedema to lymphangiosarcoma. Here we introduce a simple idea with staged Charles procedure by a case report to decrease the size of wound healing in each procedure and decreasing the rate of surgical complication.

## INTRODUCTION

Lymphatic duct obstruction is a disease having long history but the treatment remained a challenge because of high recurrence rate and lack of curative methods. The goal to treat lymphedema emphasizes on limb decongestion since the underlying disease are often hard to control. The general principle to manage lymphedema always starts from conservative treatment including decongestion therapy, therapeutic exercise, skin hygiene and medication. Surgical intervention was reserved for patient’s who failed on medical treatment as in the late stage of lymphedema.

Charles procedure was first described in 1912 with radical excision of all affected skin and subcutaneous tissues down to deep fascia and coverage using split skin grafts harvested from the specimen in patients with lymphedema.^1^ It is a significant debulky surgery that some complications would be encountered like poor graft take, regrafting, massive blood loss, continuous lymphatic fluid oozing and postoperative infection.^2^ The cosmetic result was also unsatisfactory. Several modified methods have been discussed.

Here we describe the staged Charles procedure splitting one stage to multiple stages surgery. In immune compromised patients, patients with unfavorable wound healing condition or patients who could not bear this operation staged Charles procedure could be an alternative way though it may prolong the treatment course.

## CASE REPORT

**Fig.1:**
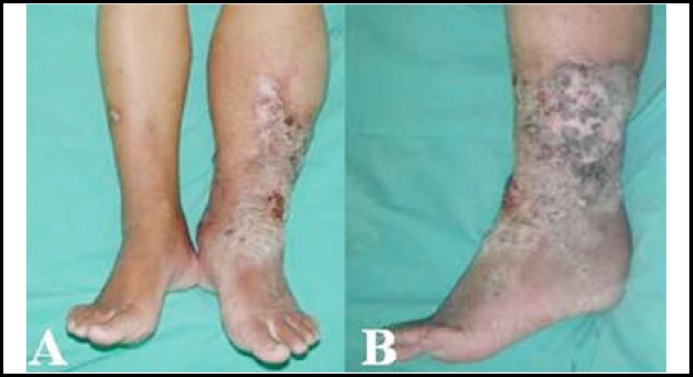
On physical examination, his left leg presented multiple ulcerative, hyperkeratotic lesions with thick, fibrotic, hyperpigmented skin discoloration (A,B).

The patient was a 46-year-old male with type 2 diabetes on oral hypoglycemic agents for 4 years. His blood sugar control was not satisfactory because of poor compliance and the HbA1C was 10.65% checked one month ago. He sustained left lower leg swelling with ulcers and repeated cellulitis for 1-2 years. During this period he was treated at the outpatient department with both oral antibiotics and wound care. In recent months the number of ulcers increased markedly with more serum-like drainage. On physical examination, his left leg had multiple ulcerative, hyperkeratotic lesions with thick, fibrotic, hyperpigmented skin discoloration (Fig.1).

**Fig.2:**
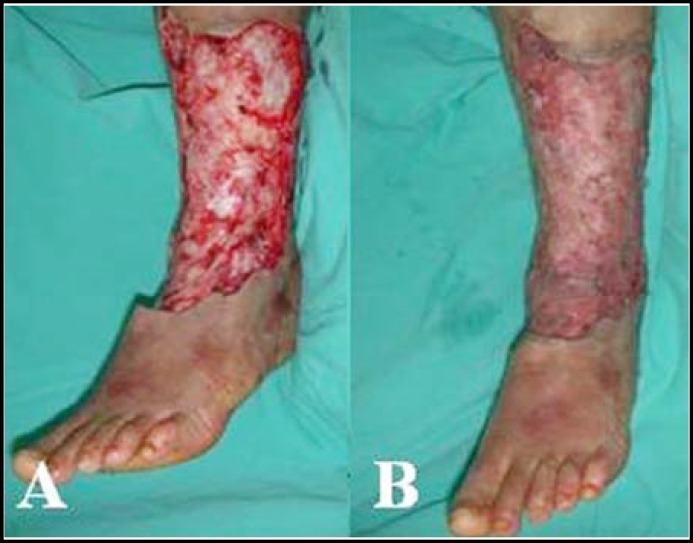
The affected skin and subcutaneous tissue was excised deep to fascia level (A) and covered with split-thickness skin graft (B).

These lesions were unilateral occurance and the involved area was about 600 cm2 from the pretibial region to the posterior aspect of leg. Significant greater circumferential distance of left lower leg was also noted with 6 cm and 10 cm longer than right lower leg in the calf and ankle level respectively. We arranged operation for the patient under the impression of unilateral lymphedema, left lower limb and diabetic foot ulcers with repeated cellulitis. Skin malignancy was suspected due to the rapid progression of these lesions. First, multiple skin biopsy was done to rule out skin malignancy and fortunately the pathology was ulcer with acute necrotizing inflammation. Second, the peripheral arterial disease was excluded by computed tomography angiography. Then, half of affected skin and subcutaneous tissue was excised deep to fascia level and covered with split-thickness skin graft (Fig.2).

**Fig.3:**
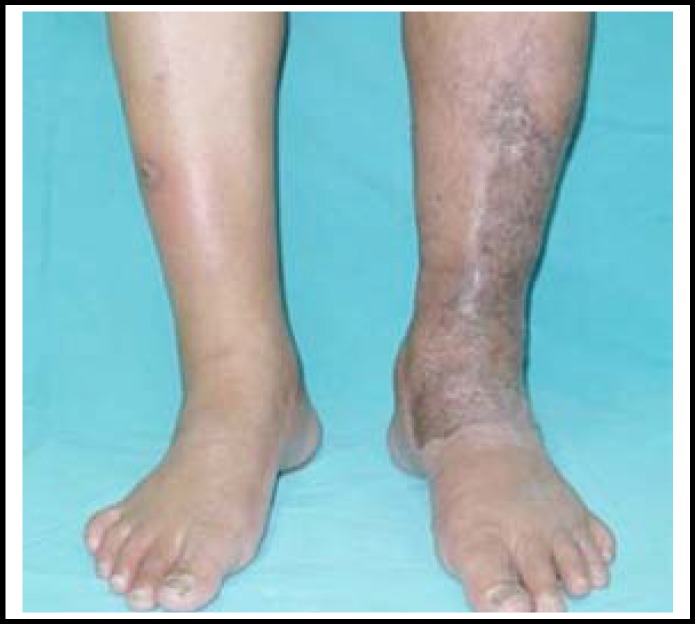
After one-year follow-up, no recurrent infection occurred and the patient was satisfied with the cosmetic results

The same procedure toward the other half lesions was completed a week later. There was no immediate post-operative complication such as blood loss requiring transfusion, failure taking of skin graft or wound infection. Combination therapy with antibiotics use and hyperbaric oxygen was also prescribed. The patient was discharged seven days after operation and compression garment was applied continuously after wound healing. In one-year follow-up, no recurrent infection occurred and the patient was satisfied with the cosmetic results (Fig.3). The difference of circumferential distance between two legs decreased to 2 cm and 1 cm in the calf and ankle level respectively.

## DISCUSSION

Lymphedema is defined as deficiency in the lymphatic system with protein-rich fluid accumulated in interstitial space. It is an old disease with variant causes and still lack in ideal resolution. Primary lymphedema is a rare disorder and often related to gene defect.^3^ It is representative of lymphatic malformation and typically classified by age of onset including congenital lymphedema, lymphedema praecox and lymphedema tarda. The incidence of primary lymphedema in those attending lymphedema clinics ranges from 8 percent among all newly diagnosed patients to 28 percent of those with non-cancer related disease.^4^ Primary lymphedema differ from secondary lymphedema with higher incidence usually which results from lymphtic disruption or obstruction. In developed countries, cases of secondary lymphedema have increased with the cancer surgery involving axillary or groin lymph nodes dissection and associated therapy, while filariasis infection remains the main etiology in developing countries.^5^

Recurrent cellulitis could be both the cause and complication of secondary lymphedema, as in our case. Repeated infection and inflammation may destroy the lymphtic vessels, leading to lymphedema, and in patients with lymphedema, the protein-rich lymph serve as a medium providing microbial proliferation. Besides repeated skin infection and affecting quality of life, another rare but severe complication of chronic lymphedema was the malignant transforming to lymphangiosarcoma. It is classically seen in the postmastectomy, lymphedematous arm (Stewart-Treves syndrome) but has also been reported in primary lymphedema and chronic filarial lymphedema. The tumor has very poor prognosis and the only treatment is amputation.

In principle, surgical therapy was indicated for the patients who fail on medical therapy. There are mainly two type of surgery: bypass surgery and resection procedure. Bypass surgery creates anastomosis between lymphatic ducts, lymphatic duct or nodes and vein. Excellent results were ever reported^6^ but in fact, the results were unpredictable in most studies and the outcome was difficult to assess. Compression garment always play an import role in the post-operative assistance and no current available methods can evaluate lymphatic vessel patency. Bypass surgery is more indicated for early stage lymphedema and became popular in several Asian countries recently because it attempts to correct the underlying disorder directly.

Charles procedure was first described in 1912 with radical resection all affected skin and subcutaneous tissues down to deep fascia with coverage using split-thickness skin grafts harvested from the excised specimen.^1^ Because it focuses on removal of the excess skin and soft tissue instead of correcting the underlying pathology, the edema may return. Charles procedure is a significant debulky surgery and some complication may develop after this procedure, including massive blood loss that needs transfusion, poor graft patency that needs regrafting, continuous lymphatic fluid oozing, postoperative infection, unsatisfactory cosmetic result, hypertrophic scar, papillomatosis, recurrent cellulitis, and lymphatic fistula. Several modified methods have been described such as delayed grafting, the donor site choosing, combination use with negative pressure dressing,^3^ and excision with preservation of perforators.^7^ Staged Skin and Subcutaneous Excision is an effective option but not favored by patients having multiple skin lesions.^8^

Our patient presented in stage III lymphedema (irreversible skin change, fibrosis, papillae) with potential risk of poor wound healing from poor control of diabetes and repeated infection. We performed two staged Charles procedure with the aim of decreasing the size of wound healing in each procedure and decreasing the rate of surgical complication. It is a simple idea but indeed decreases the risk of blood loss, area of skin graft failure and can be a predictor of outcome of next procedure. If the area needs to be resected is large, the procedure can be divided in to three or more stages. In immunocompromised patients, patients with unfavorable wound healing condition or patients who could not bear this massive volume reduction surgery, staged Charles procedure provided an alternative method that obtained same or better results.

Modifying the Charles procedure by two-stage skin grafting results in a clean dry bed that accepts grafts readily. Skin graft loss is detrimental for patients undergoing the Charles procedure. Another advantage of applying a staged Charles procedure immediately postoperatively was the improved ease of postoperative care. Staged Charles procedures are well tolerated and result in a satisfactory and predictable outcome. We feel that this simple modification makes this procedure easier to perform, it leads to minimal immediate complications and it results in a satisfactory outcome for patients although long-term studies are needed to compare the long-term outcome.

## Notes

***Financial Disclosure:*** None of the contributing authors have any conflicts of interest, including specific financial interests and relationships and affiliations relevant to the subject matter or materials discussed in the manuscript.
